# The P4-phospholipid flippase Atp11a is required for maintenance of eye and ear structure in zebrafish

**DOI:** 10.1242/jcs.263657

**Published:** 2025-05-22

**Authors:** Alexia Hawkey-Noble, Cameron Tobin, Muhammad T. Ameen, Liam Osmond, Colby Gill, Christina S. Bottaro, Terry-Lynn Young, Curtis R. French

**Affiliations:** ^1^ Division of Biomedical Sciences, Faculty of Medicine, Memorial University of Newfoundland, St John's NL A1B 3V6, Canada; ^2^ Department of Chemistry, Faculty of Science, Memorial University of Newfoundland, St John's NL A1C 5S7, Canada

**Keywords:** *atp11a*, Zebrafish, Photoreceptor, Retinal pigment epithelium, Hair cell, Stereocilia

## Abstract

The *atp11a* gene encodes a phospholipid flippase protein required to flip phosphatidylserine (PS) and phosphatidylethanolamine (PE) from the outer leaflet of the cytoplasmic membrane to the inner leaflet. Mutations in *ATP11A* have been described in individuals with sensorineural hearing loss and neurological deterioration; however, little is known regarding the mechanism by which loss of *atp11a* results in such phenotypes. To this end, we created loss-of-function *atp11a* mutant zebrafish to characterize potential disease states. We demonstrate that mutant *atp11a* zebrafish display a reduced number of stereocilia in the larval ear and a reduced number of hair cells in some sensory neuromasts, indicating that these fish represent an ideal model for studying *atp11a*-attributable hearing loss. In addition, *atp11a* mutant zebrafish raised in a standard light cycle have reduced photoreceptor outer segments, the severity of which is lessened when mutant larvae are raised in the dark. Photoreceptors that do remain in homozygous *atp11a* mutants undergo mitochondrial fission and produce an increased number of mitochondria, suggesting that defects in energy homeostasis may contribute to or result from outer segment degradation.

## Teaser

**Highlighted Article:** Mutation of *atp11a* in zebrafish causes photoreceptor degradation and defects in the ear and lateral line neuromast system.

## INTRODUCTION

Phospholipid membrane asymmetry is an essential component of cellular function and is conserved among almost all forms of prokaryotic and eukaryotic cells. Pioneering studies decades ago established that eukaryotic cells sequester the majority of their phosphatidylethanolamine (PE) and the negatively charged lipids phosphatidylserine (PS) and phosphatidylinositol (PI) in the inner cytoplasmic side of the plasma membrane, while sphingomyelin (SM) and phosphatidylcholine (PC) are found primarily on the outer leaflet (Devaux, 1991; Verkleij et al., 1973). This asymmetry helps to establish membrane properties such as resting potential, shape, permeability and stability (Devaux, 1991), and might contribute to the formation of functional domains within the membrane, such as lipid rafts (Cheng et al., 2009). In mammals, changes to the maintenance of membrane asymmetry, such as the exposure of PS on the outer membrane leaflet, are required for blood coagulation and clearance of damaged cells by phagocytic macrophages (Lentz, 2003; Majumder, 2022; Segawa and Nagata, 2015; Vorselen, 2022).

As phospholipids are randomly inserted in the cell membrane, asymmetry is established and maintained through the flipping of phospholipids from one leaflet to the other using an energy-dependent process. The ‘flippases’ are a family of P_4_ ATPases that hydrolyze ATP to flip phospholipids from the outer to the inner leaflet, whereas a family of ABC transporters known as ‘floppases’ transport lipids in the opposite direction. An additional family of scramblase proteins randomly flip phospholipids between each leaflet in an energy-independent process, and are, for example, required for the exposure of PS on the outer leaflet of the membrane during normal blood clotting in mammals (Hankins et al., 2015; Suzuki et al., 2010). Five classes of flippases based on the substrate type are present in most mammalian cells, including class 1A (ATP8A1-2), class 1B (ATP8B1-5), class 2 (ATP9A and ATP9B), class 5 (ATP10A, ATP10B and ATP10D) and class 6 (ATP11A-C).

Defects in these phospholipid flippase and floppase proteins have been linked to human disease. For example, mutations in *ATP11A* have been found in families with sensorineural hearing loss inherited in an autosomal dominant fashion (Chepurwar et al., 2023; Pater et al., 2022; Vona et al., 2023), while variants near this gene have been linked to severe Covid19 through genome-wide association studies (GWAS) and splicing quantitative trait loci (sQTL) analysis (Kousathanas et al., 2022; Nakanishi et al., 2023). Mutations in *ATP8B1* can cause intrahepatic cholestatic disorders (Bull et al., 1998; Minguez Rodriguez et al., 2022; Piazzolla et al., 2020), while mutation of *ATP8A2* causes a number of cerebellar ataxia-based syndromes (Guissart et al., 2020; Narishige et al., 2022). Mutation of *ATP9A* causes autosomal recessive neurodevelopmental disorder with poor growth and behavioral abnormalities (NEDGBA) (Mattioli et al., 2021; Vogt et al., 2022), and loss of *ATP11C* cause hemolytic anemia (Arashiki et al., 2016, 2019). Loss of membrane asymmetry has been documented in cancer cells and has been linked to changes in membrane permeability that facilitate resistance to chemotherapy (Miyoshi et al., 2010; Zhang et al., 2005).

Animal models have provided additional insight to the role of P4-flippases in health and disease. Mouse models that encode a loss-of-function *Atp1lc* mutation show an induced cholestasis phenotype mimicking that in individuals with mutations in *ATP8B1* (Siggs et al., 2011). *Atp8a2* mutation results in eye and ear defects in mice, with degeneration of the ocular photoreceptors and the otic spiral ganglion cells noted (Coleman et al., 2014). Expression of a similar P4-Atpase, *atp11a*, is found in the zebrafish eye and ear suggesting similar functions (Hawkey-Noble et al., 2020). As mutation of *ATP11A* results in sensorineural hearing loss in human pedigrees (Chepurwar et al., 2023; Pater et al., 2022; Vona et al., 2023) we sought to create an *atp11a* mutant zebrafish to model human deafness. Additionally, given its expression in the outer layer of the eye in zebrafish, we sought to decipher the role of this phospholipid flippase in ocular development and maintenance of photoreceptor health.

## RESULTS

### *atp11a* mutants are hypopigmented and die before sexual maturation

**Fig. 1.**
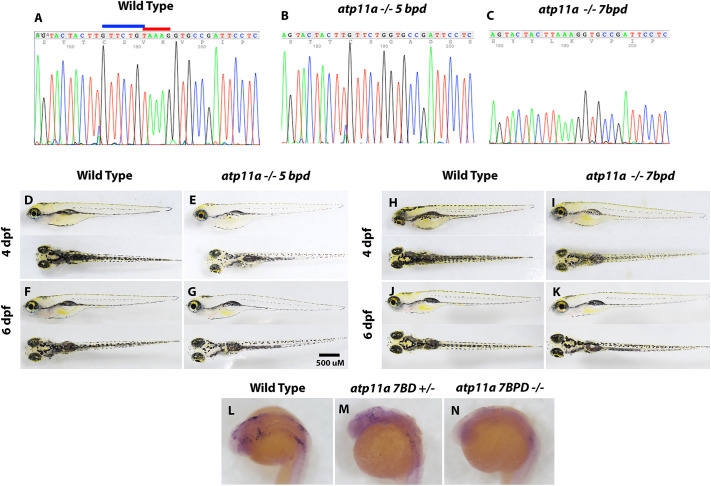
**Generation of *atp11a* loss-of-function mutants**. (A–C) Two mutant strains were isolated using the same gRNA, creating a 7 bp deletion (GTTCTGT) highlighted by the blue bar and a 5 bp deletion (TAAAG) highlighted by the red bar. (D–K) At both 4 and 6 dpf, larvae are morphologically wild type, inflate their swim bladders and have reduced pigment. (L–N) *In situ* hybridization using an antisense *atp11a* probe demonstrates reduced transcript levels in heterozygous and homozygous mutant embryos at 24 hpf.

We used CRISPR gene editing targeting a guide RNA to exon 17 (ENSDARE00000252474) of the principal isoform of the *atp11a* transcript to create a potential loss-of-function mutation. Exon 17 encodes a HAD-like super family domain common to ATPase proteins and is present in all annotated protein-coding transcripts. Two mutant strains were created and used in the current study, one containing a 5 bp deletion (*atp11a^nl1005^*) and the other containing a 7 bp deletion (*atp11a^nl1007^*) in exon 17 (Fig. 1A–C). Heterozygous in-crosses for both strains produce the expected genotypes with correct Mendelian ratios in early larval stages. Homozygous mutants inflate their swim bladders and survive until at least 6 days post-fertilization (dpf) (Fig. 1D–K); however, we have not recovered any homozygous adults at sexual maturity, based on genotyping of 16 *atp11a^nl1005^* and 24 *atp11a^nl1007^* larvae, resulting from pairwise in-crosses (data not shown). *In situ* hybridization demonstrates a reduction in *atp11a* expression in homozygous mutants (Fig. 1L–N), indicating nonsense mediated decay of mutant transcripts. Loss of *atp11a* in these strains results in hypopigmented embryos (Fig. 1D–K), with a reduction in pigment coverage quantified in the head (57% reduction, *P*=0.027, Fig. S1).

### Reduced numbers of stereocilia in the ears of *atp11a* mutants

**Fig. 2.**
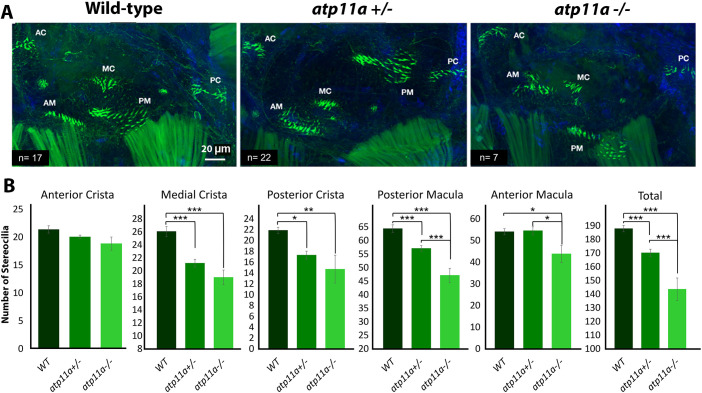
**Reduced number of stereocilia in *atp11a* mutants**. (A) Phalloidin staining of F-actin filaments in the hair cells of the *atp11a^nl1005^* zebrafish inner ear at 5 dpf, illustrating significant hair cell loss in the medial crista (MC), posterior crista (PC) and macula (PM), as well as the anterior macula (AM), resulting in an overall decrease in the inner ear. (B) Hair cell quantification for the anterior crista (AC), anterior macular, medial crista, posterior crista and posterior macula demonstrates a reduction in stereocilia in heterozygous and homozygous mutants. Data are mean±s.e.m. with significance testing (*P<0.05, **P<0.01, ***P<0.001) calculated using a one-factor ANOVA with Tukey's post-hoc analysis for multiple comparisons. Wild type, *n*=17; heterozygous, *n*=22; homozygous mutant, *n*=7.

To assess hearing-related phenotypes in *atp11a* mutants, we assessed the ear and lateral line hair cells, which both regulate hearing and balance in zebrafish. The larval zebrafish ear contains five patches of sensory epithelium containing stereocilia that are responsible for turning mechanical stimulation into electrical signals to be interpreted by the brain (McGrath et al., 2017; Whitfield, 2020). Numerous zebrafish deafness models display defects in these structures (Han et al., 2018; Vona et al., 2021). A reduction in stereocilia number, as evidenced by phalloidin staining, was observed in the medial crista, posterior crista, posterior macula and anterior macula of homozygous *atp11a* mutants (Fig. 2A,B). Intermediate phenotypes, that were also statistically significant, were observed in heterozygous animals.

**Fig. 3.**
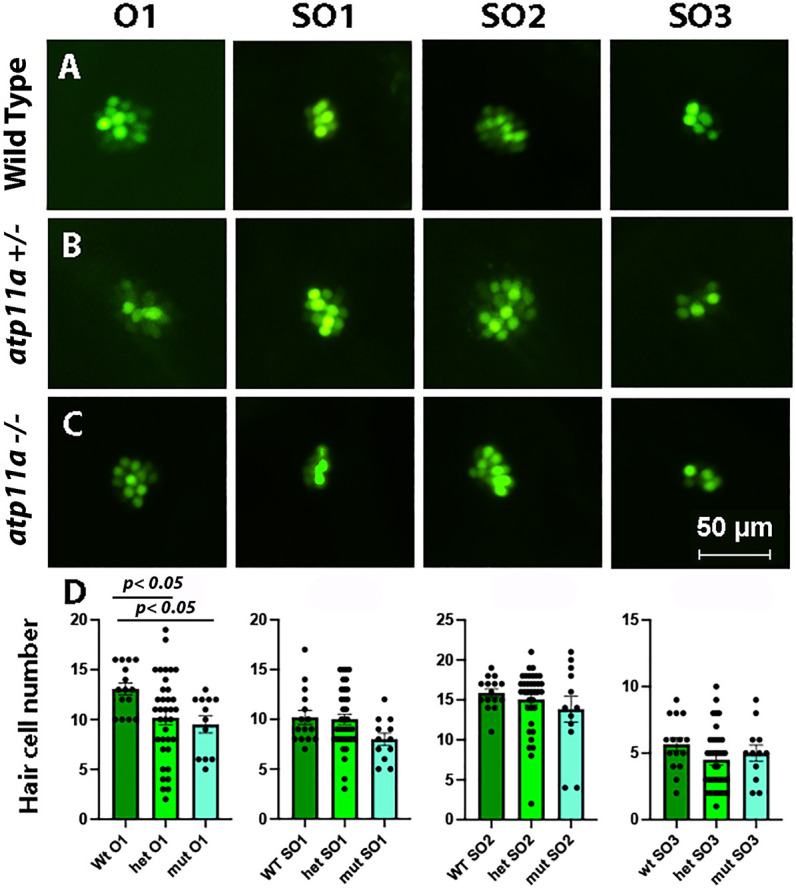
**Reduced number of hair cells in the O1 neuromast**. (A–C) Nuclear staining of four neuromasts in 5 dpf *atp11a^nl1007^* larvae with Yo-Pro-1, demonstrating a decrease in the number of positively stained cells in the O1 neuromast in homozygous and heterozygous mutants compared with wild type. (D) Neuromast hair cell quantification for one otic (O1) and three supraoptic (SO1, SO2 and SO3) neuromasts demonstrates a significant reduction in hair cell number for the O1 neuromast in homozygous and heterozygous mutants. Data are mean±s.e.m. with significance calculated using a one-factor ANOVA with Tukey's post-hoc analysis for multiple comparisons. Wild type, *n*=15; heterozygous, *n*=36; homozygous mutant, *n*=12.

We next assessed the number of lateral line neuromast hair cells for viability and the nerve innervation required for sensing changes in water currents. Using the nuclear stain Yo-Pro-1, which brightly stains neuromast hair cell nuclei (Ou et al., 2012), we observed a reduction in the number of hair cells in the O1 neuromast in both homozygous and heterozygous mutant larvae; however, analysis of other neuromasts (SO1, SO2 and SO3), showed no phenotype compared to wild-type siblings (Fig. 3A–D). The uptake of the stain (DASPEI) via metabolically active mitochondria in the neuromasts was used to assess neuromast hair cell viability, and no difference was observed between *atp11a* mutant and wild-type siblings (Fig. S2A–C,G). Analysis of nerve innervation (afferent fibers) and hair cell number in the lateral line neuromasts was assessed using an anti-Hnk1 antibody; again, no differences were noted between *atp11a* mutants and wild-type siblings (Fig. S2D-F,H).

### Loss of photoreceptors in *atp11a* mutant eyes

**Fig. 4.**
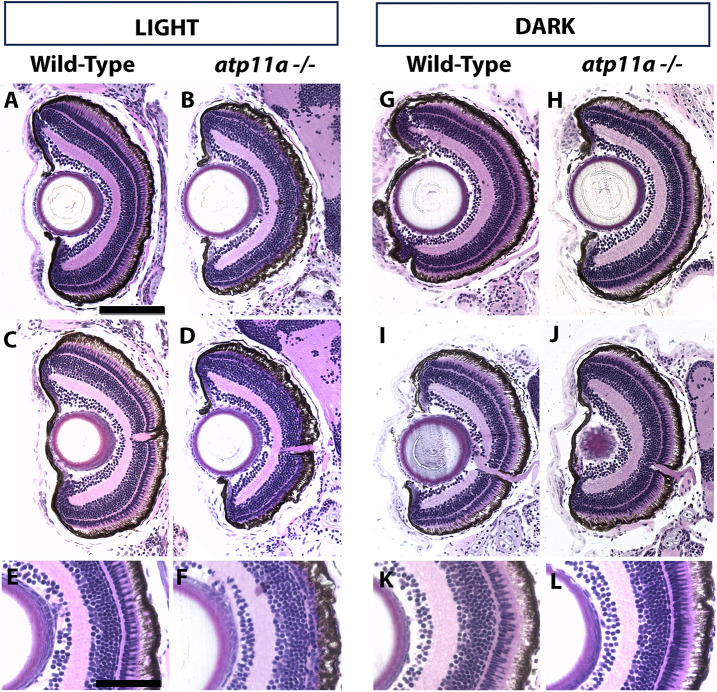
**Photoreceptor loss in *atp11a^nl1007^* mutants is partially light dependent**. (A,C,E) Wild-type larvae display regular retinal lamination with a distinct photoreceptor layer. (B,D,F) *atp11a^nl1007^* larvae raised in ambient light display a reduced photoreceptor layer. (G–L) When raised in the dark, both wild-type (G,I,K) and *atp11a^nl1007^* larvae (H,J,L) have a more wild-type appearing photoreceptor layer. Wild type light, *n*=9; homozygous mutant light, *n*=7; wild type dark, *n*=8; homozygous mutant dark, *n*=9. All larvae are at 6 dpf. Scale bars: 100µm in A for A–D,G-J; 50 µm in E for E,F,K,L.

**Fig. 5.**
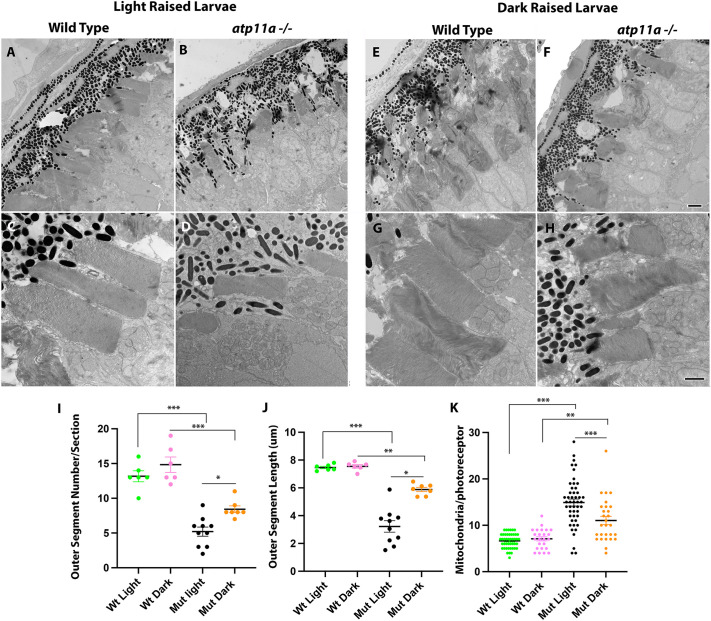
**Ultrastructure analysis of *atp11a^nl1007^* mutant photoreceptors.** (A–D) Photoreceptors in ultrathin sections show a reduction of outer segments in homozygous mutants when compared to wild-type siblings. (E–H) This phenotype partially resolves when mutant larvae are raised in the dark. Quantification of outer segment number (I), length (J) and mitochondrial number (K) confirm a partial rescue via dark-rearing conditions. Data are mean±s.e.m. ***P<0001, **P<0.001, *P<0.01 (one-factor ANOVA with Tukey’s analysis for multiple comparisons). Wild type light, *n*=6; mutant light, *n*=6; wild type dark, *n*=3; mutant dark, *n*=3. Larvae are at 6 dpf. Scale bars: 2 µm in F for A,B,E,F; 1 µm in H for C,D,G,H.

Given the expression of *atp11a* in the retina in zebrafish and mice (Hawkey-Noble et al., 2020; Wang et al., 2018), we assessed retinal lamination in eye sections at 6 dpf. Loss of *atp11a* results in a reduction of the photoreceptor layer (Fig. 4A–F). Some variability exists, with some patches of retina appearing severely affected, while others appear more subtly affected (Fig. S3). Transmission electron microscopy (TEM) revealed photoreceptor outer segments that are entirely lost or reduced in size (Fig. 5A,B), while their inner segments have an increased number of mitochondria (Fig. 5C,D). Again, there is considerable variability, with some sections displaying a more severe phenotype than others (Fig. S4).

The loss of photoreceptor outer segments could be due to a defect in their intrinsic development or, alternatively, outer segments could be formed normally and degraded due to defects in disc phagocytosis, recycling or a block in the visual cascade. To add insight into these possibilities, we raised zebrafish larvae in the dark beginning at 24 hpf. In the absence of light, *atp11a* mutants display reduced severity of the photoreceptor outer segment phenotype with clear inner and outer segments (Fig. 4G–L), similar their wild-type siblings, as opposed to the reduced outer segment layer observed when mutant larvae are raised in a standard light cycle. This is confirmed using TEM analysis, as dark-raised larvae show reduced severity of outer segment degeneration (Fig. 5E–H; Fig. S4). Dark-raised mutant larvae display a partial rescue, where increased numbers of outer segments, increased length of outer segments and an increased number of mitochondria are observed compared to light-raised mutants. However, the dark-raised larvae are still significantly different from wild-type larvae (Fig. 5I–K).

### Light-independent cell death occurs in *atp11a* mutant eyes

**Fig. 6.**
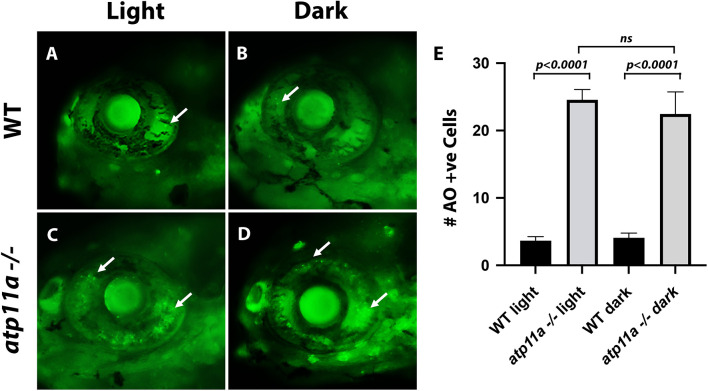
**Cell death in *atp11a^nl1007^* mutant eyes.** (A,B) Few dying cells (Acridine Orange positive, arrows) are observed in wild-type sibling eyes in light and dark conditions. (C,D) In *atplla* homozygous mutants, an increase in the number of Acridine Orange-positive cells (arrows) is observed in light and dark conditions. (E) Quantification of cell death (number of Acridine Orange-positive cells), presented as mean±s.e.m. Wild type light, *n*=9; homozygous mutant light, *n*=9; wild type dark, *n*=10; homozygous mutant dark, *n*=11. Significance testing was carried out using two-factor ANOVA. All larvae are at 6 dpf.

We next assessed whether cell death was occurring in *atp11a* mutant eyes given the known role of Atp11a in apoptosis and phagocytosis of dying cells (Nagata, 2018; Nagata and Segawa, 2021). At early developmental time points before the onset of *atp11a* expression in the eye (2 dpf), no cell death over baseline was observed (Fig. S5A,B,E). At 3 dpf, the time at which photoreceptors become functional and *atp11a* expression is observed in the outer ocular layers (Hawkey-Noble et al., 2020), increased cell death is observed in mutant eyes (Fig. S5C,D,F) and a further increase is observed at 5 dpf (Fig. 6A–E). Raising larvae in the dark has no effect on cell death levels, as homozygous mutants still displayed increased cell death compared to wild-type siblings raised in standard a light cycle. (Fig. 6B,D,E).

### Behavioral defects in *atp11a* mutants

**Fig. 7.**
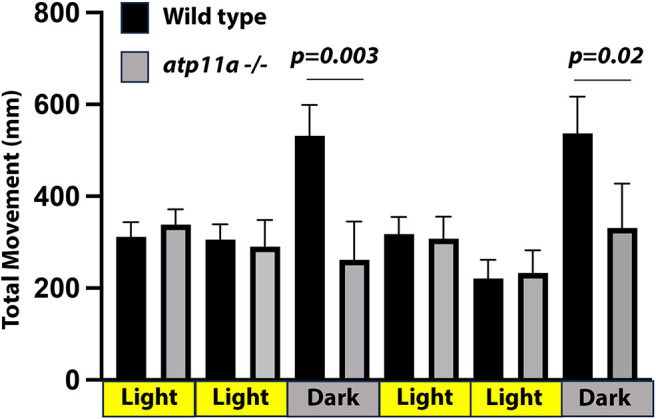
***atp11a* mutants have altered swimming behavior.** When transitioning from light to dark, wild-type larvae have a stereotypical increase in swimming activity, which is not observed in *atp11a^nl1007^* mutant larvae. Wild type, *n*=11; mutant, *n*=7. Data are mean±s.d. Significance testing was carried out using a one-factor ANOVA with Tukey's analysis of multiple comparisons. Analysis was performed at 7 dpf.

To test visual perception- and hearing loss-related behaviors in *atp11a* mutants, we analyzed swimming characteristics in *atp11a* mutants and wild-type siblings. It has been demonstrated that zebrafish larvae increase movement when changing from light to dark conditions, and then reduce their movement upon returning to light (Ellis et al., 2012; Tan et al., 2022). This behavior was observed in wild-type siblings, but not in *atp11a* homozygous mutants who did not alter their movement patterns between light and dark conditions (Fig. 7), indicating a potential visual defect in these larvae. Analysis of circular swimming behavior, often observed in zebrafish deaf mutants (Nicolson et al., 1998), did not show any differences between wild type, heterozygous or homozygous mutants in light or dark conditions (Fig. S6).

## DISCUSSION

In this work, we describe two previously unreported zebrafish strains, containing loss-of-function mutations in the *atp11a* gene, as having defects in pigmentation, ear development and a light-induced degeneration of photoreceptors. This agrees with our previous work detailing expression of *atp11a* in neural crest-derived pigment cells, the ear and the outer layers of the eye (Hawkey-Noble et al., 2020). Phenotypes are present in two different alleles of *atp11a* (*atp11a^nl1005^* and *atp11a^nl1007^*), and are thus likely attributable to *atp11a* loss of function as opposed to any off-target effects of CRISPR mutagenesis.

Loss of hair cell stereocilia in the ear is observed in *atp11a* mutant zebrafish, in agreement with hearing loss in human individuals and mouse models (Pater et al., 2022; Vona et al., 2021, 2023). While there is no evidence for changes to innervation of neuromasts in *atp11a* mutants, the O1 neuromast located near the ear demonstrated differential uptake of the Yo-Pro-1 stain. This stain has recently been shown to be an indicator of mechanotransduction capacity (Patterson et al., 2024); thus, this result could indicate cell loss or loss of mechanotransduction capacity. These cells coordinate with the ear to contribute to vestibulocochlear functions in zebrafish. Circling behavior is often exhibited by animals exhibiting hearing loss phenotypes, including mice and zebrafish (Lee et al., 2001; Nicolson et al., 1998). Yet despite evidence for the disruption of structures related to auditory function, there was no difference in the number of circles swum by *atp11a* mutants when compared to their wild-type or heterozygous siblings. It is possible that other flippases, such as *atp11b*, which is also expressed in the zebrafish ear (Hawkey-Noble et al., 2020), may be able to partially compensate for the loss of *atp11a* and allow for normal swimming. However, given the synteny of zebrafish *atp11a* with human *ATP11A* (Hawkey-Noble et al., 2020), and the observed defects in ear and some neuromast hair cells, zebrafish *atp11a* mutants make an reasonable model for further work to address mechanisms of hearing loss due to ATPllA dysfunction in human individuals. Further work will be needed, such as the testing of specific audio frequencies for the production of audiograms (Yao et al., 2016), to determine if there are specific aspects of hearing loss that occur in zebrafish *atplla* mutants that align with patient-based phenotypes.

Within the eye, the degeneration of photoreceptor outer segments is evident in mutant larvae raised in a standard light cycle but is reduced in larvae raised in the dark. This could imply a defect in the RPE that expresses high levels of *atp11a* (Hawkey-Noble et al., 2020). It is possible that the RPE is unable to recycle outer segment discs after engulfment, which would be blunted by the absence of light as this would reduce the phagocytosis of disc membranes via the RPE. The cell death observed in the outer eye could represent the death of retinal pigment epithelium cells that could impact the recycling of photoreceptor discs or the photopigment itself, as seen in zebrafish *rep1* mutants where a cell non-autonomous loss of photoreceptor outer segments occurs via interaction with mutant RPE cells (Krock et al., 2007). Defects in vesicle trafficking, required to add recycled materials back to the outer segment discs, could also play a role but were not tested as part of this study.

Alternatively, there are specific cell-autonomous roles for PS and PE in the photoreceptors that could impact the health of these cells. Exposure of PS on the outer leaflet of the plasma membrane is a signal for phagocytosis of the discs by the RPE (Moran et al., 2022; Ruggiero et al., 2012); thus, an inability to remove PS from the outer leaflet in an *atp11a* mutant background may result in the phagocytosis of outer segment discs, regardless of whether or not they need to be recycled. As exposure of light leads to increased PS exposure (Ruggiero et al., 2012), the reduction of disc degeneration in larvae raised in the dark also supports this hypothesis. PE is also required in the visual cycle, whereby it is required to bind all-trans retinal for removal from the photoreceptor discs after its light-induced conversion from 11-cis retinal. The inability to remove all-trans retinal, seen in Stargardt's disease, leads to photoreceptor degeneration. Stargardt's disease is caused by mutation in the phospholipid floppase gene *ABCA4*, which is required of removal of all-trans retinal-PE complexes (Molday et al., 2009; Tsybovsky et al., 2010); thus, loss of *atp11a* may result in the decreased availability of PE on the inner membrane leaflet, decreasing the ability to remove all-trans retinal through Abca4. However, further work will be required to distinguish the precise involvement of PS or PE, and the role of RPE recycling in *atplla*- and light-attributable photoreceptor degeneration.

Photoreceptors that do remain in *atp11a* mutant retinas have an increase in the number of mitochondria in their inner segments. It is known that zebrafish and other vertebrates undergo mitochondria fission in their photoreceptors as part of the natural aging process (Burgoyne et al., 2022), whereby mitochondria split in order to increase their efficiency at generating ATP in response to accumulated photoreceptor damage. Thus, it is possible that the increased number of mitochondria observed in the remaining *atp11a* mutant inner segments is a response to potential visual defects, although a causative effect on photoreceptor health cannot be ruled out given an increased number of mitochondria are still observed in dark-raised larvae. Mitochondria help to direct light to the outer segments of photoreceptors (Ball et al., 2022), so increased numbers of mitochondria could result in increased light absorption via the photoreceptor outer discs and an increased rate of phagocytosis.

Defects in photoreceptor function and retinal neuron survival in *atp11a* mutants may lead to visual and behavioral defects. It has been established that zebrafish increase their activity when transitioning from light to dark conditions (Ellis et al., 2012; Tan et al., 2022). Although suggested as a stress response, this change in behavior requires the larvae to distinguish between light and dark. Homozygous *atp11a* mutants fail to increase their activity in response to changing from light to dark conditions, which could indicate a visual defect. However, it is not clear whether the light and dark behavioral responses are sensed via the photoreceptive cells of the retina or the pineal gland (which also contains photoreceptors and is involved in circadian rhythm regulation in zebrafish); thus, further testing will be required to definitively determine if vision is negatively altered in *atp11a* mutant zebrafish. Cell death is observed in the eye of *atp11a* mutants but it is not clear what cells may be dying in the eye and whether this contributes to the potential loss of visual function. Given that the cell death is not rescued in dark raised larvae, it is unlikely related to the photoreceptor degeneration phenotype.

Zebrafish *atp11a* mutants were created to model deafness, given the description of four families with hearing loss segregating with *ATP11A* mutations. To our knowledge, none of the families report defects in vision, possibly highlighting differences between human and zebrafish ATP11A and Atp11a function. However, the described genetic lesions in human individuals are all splice site mutations, and their effect on gene function is not entirely known. In one family, the splice site mutation involves a rare exon that is present in a minority of transcripts and does not include the annotated principal isoforms (Pater et al., 2022). While these human individuals might develop vision loss in the future, it is possible that the eye defects observed in zebrafish mutants may be the result of a CRISPR-induced loss-of-function mutation and that such phenotypes may not manifest in human individuals with splice site mutations that do not affect all known transcripts. Alternatively, such human individuals may not exhibit ocular defects because they harbor heterozygous *atp11a* mutations. Although we observe defects in ear hair cells and the O1 neuromast in heterozygous *atp11a* fish, ocular defects are only observed in homozygous mutants. *Atp11a* is expressed in the mouse retina (Wang et al., 2018) and while no eye abnormalities have been reported in *Atp11a* mutant mice, this might not have been tested specifically (Ochiai et al., 2022). The eye and ear defects in *atp11a* mutant zebrafish are similar to those reported in *Atp8a2* mutant mice, which also display ear defects and reduced photoreceptor outer segments (Coleman et al., 2014), possibly highlighting functional homology between these two related P4-ATPases.

In summary, loss of *atp11a* gene function in zebrafish demonstrates a conserved function for this phospholipid flippase in the ear between humans and zebrafish, as defects in the ear and the O1 neuromast are observed in zebrafish mutants. Additional phenotypes, including loss of photoreceptor outer segments and increased cell death in the eye highlight additional roles for membrane asymmetry, and its maintenance via Atp11a in the eye. Amelioration of photoreceptor outer segment loss in dark-raised larvae indicates that ocular defects are at least partially due to extrinsic factors.

## MATERIALS AND METHODS

### Zebrafish husbandry

All zebrafish were raised under standard conditions and staged as hours or days post-fertilization (hpf and dpf, respectively), as previously described (Kimmel et al., 1995). This study was conducted in compliance with the requirements and regulations defined by the Animal Care Committee of Memorial University of Newfoundland and the Canadian Council on Animal Care. For experiments involving the ear, 0.003% 1-phenyl 2-thiourea (PTU; Sigma-Aldrich) was used in standard embryo medium (Nüsslein-Volhard and Dahm, 2002) to prevent pigmentation on all embryos 24 hpf and older to maintain optic clarity. Embryos were fixed using 4% paraformaldehyde (PFA; Sigma-Aldrich) and, where necessary, for embryos 48 hpf and older, 0.168 mg/ml tricaine was used as an anesthetic.

### Generation of *atplla* mutants

Alt-R crRNA (3 μl) with the sequence GAGTACTACTTGTTCTGTAA, targeting exon 17 of the principal *atp11a* transcript (ENSDART00000150029.3), was used to generate stable mutant strains. Targeting this region not only ensures the mutation of all possible transcripts that could be expressed but simultaneously targets the functional domain involved in the flipping of phospholipids. crRNA was incubated for 5 min with a tracrRNA in Duplex Buffer (IDT) for 5 min at 95°C and then added to CAS9 buffer consisting of 20 mM HEPES; 150 mM KCl (pH 7.5) and CAS9 protein. The mixture was then heated to 37°C for 10 min to assemble ribonucleotide-protein complexes. The final injection mixture contained 18 ng/µl crRNA, 33.5 ng/ult rRNA and l.025 µg/l CAS 9 protein. Approximately 3 nl was injected into one-cell zebrafish embryos. Two mutant lines were generated for this study via outcross of mosaic F0 mutants to wild-type (AB) strains; one mutant line was generated with a 5 bp deletion in exon 17 (designated *atp11a^nl1005^*) and another with a 7 bp deletion in exon 17 (designated *atp11a^nl1007^*).

### *In situ* hybridizations

*In situ* hybridizations were carried out using previously published protocols (Thisse and Thisse, 2008). The *atp11a* probe was generated as outlined previously (Hawkey-Noble et al., 2020) and used on *atp11a^nl1007^* embryos at 24 hpf to demonstrate the degradation of *atp11a* mRNA through the nonsense-mediated decay pathway.

### Immunohistochemistry and phalloidin staining

For staining with the Hnk-1 antibody, 4 dpf *atp11a^nl1007^* larvae were permeabilized using 10 µg/ml Proteinase K and re-fixed in 4% PFA for 20 min. Embryos were washed four times for 5 min each in PBST with subsequent antigen retrieval using 10 mM sodium citrate (pH 6). Larvae were blocked in 5% normal goat serum and 2 mg/ml BSA, and incubated with the anti-Hnk-1 antibody (ZIRC) at a 1/500 dilution overnight at 4°C. Larvae were washed five times for 15 min each in PBST, incubated in goat anti-mouse Alexa Fluor 568 (Thermo Fisher Scientific) for 2 h and then washed five times for 15 min each in PBST. A Zeiss Airyscan 2 confocal microscope was used to image neuromasts.

For staining of ear hair cell stereocilia, wild-type and *atp11a^nl1005^* larvae were anesthetized and fixed in PFA at 5 dpf. Following fixation, larvae were washed with PBSTx (0.2% Triton X-100 and 1% PBS, Sigma-Aldrich) and permeabilized in 5% Triton X-100 in PBS overnight at 20°C with agitation. Larvae were then washed three times for 5 min each in PBSTx before labelling with Alexa Fluor 488 Phalloidin (1:50, Thermo Fisher Scientific) in regular PBST (0.1% Tween-20 in 1%PBS) at room temperature for 4 h in the dark with agitation. A final wash using PBSTx was performed prior to whole mounting laterally in Prolong Gold (Thermo Fisher Scientific). *Z*-stack images were taken through the entire ear using a Zeiss LSM 900 with Airyscan 2, and are presented as maximum intensity projections.

### Live stains: DASPEI, Acridine Orange and Yo-Pro1

The cell death stain Acridine Orange (AO; Sigma-Aldrich) was dissolved into embryo medium at a concentration of 10 µg/ml and applied for 1 h. Three 5-min washes with embryo medium were carried out, and the larvae were imaged using a Nikon SMZ18 microscope with a Nikon Intensilight C-HGFI exciting at 480 nm to detect positively stained cells. Upon imaging, larvae were individually transferred to 50 µl Eppendorf PCR tubes on ice for DNA extraction and genotyping.

For vital staining of neuromasts, the mitochondrial stain 2-[4-(dimethylamino)styryl]-N-ethylpyridinium iodide (DASPEI) was used at a working concentration in of 0.005% in embryo medium for 15 min, and detected with a wide-red filter after UV lamination, with neuromast quantified throughout the lateral line and head. For staining of lateral line neuromast hair cells, larvae were immersed in 2 µM Yo-Pro-1 (Invitrogen Molecular Probes) for 1 h, followed by three 5-min washes with embryo medium. Once stained, larvae were imaged using a Nikon ECLIPSE Ti2-e microscope, with fluorescent excitation at 480 nm to visualize positively stained cells. Once imaged, the individual cells for three neuromasts near the dorsal aspect of the eye and for one neuromast near the dorsal aspect of the ear were quantified, followed by DNA extraction and genotyping.

### Eye sectioning and staining

Larvae (6 dpf) were dehydrated and embedded in methacrylate resin, as previously described (Umali et al., 2019). 3 µM sections were cut through eyes using a Leica RM2165 microtome. Eyes were stained with Celestine-Blue and Eosin-Phloxine, and visualized using an Olympus Fluoview FV300 microscope with a SC50 Olympus 5 megapixel digital color camera. For analysis of eye structure under dark conditions, larvae were protected from light from 24 hpf onwards. For larvae raised in light, a light cycle of 13 h daylight/11 h dark light was used. Embryos were fixed 4 h after the lights were turned on for the day.

### Transmission electron microscopy

Samples were fixed in Karnovsky fixative for 20 min and post-fixed in 1% osmium tetroxide (15 min) in 0.1 M sodium cacodylate buffer pH 7.4 (Sigma Aldrich). Larvae were washed in sodium cacodylate buffer and then dehydrated in a graded ethanol series and embedded with Epon resin. Samples were polymerized overnight at 70°C. Ultrathin sections were cut at 80-100 nm and mounted on 300 mesh copper grids, stained with uranyLess and lead citrate (Electron Microscopy Sciences) and examined in a Tecnai Spirit TEM (FEI) operating at 80 KV.

### Pigmentation assays and quantification

For pigmentation quantification, larvae were fixed in 4% PFA after being raised in standard lighting conditions. Pictures were taken with reflected light on a white background. For quantification, a region of interest (ROI) spanning from the anterior forebrain to the posterior hindbrain was drawn using Fiji software, and the percentage of pigment coverage in the ROI was calculated using a constant threshold value for images. Samples were anonymized with genotyping performed after analysis.

### Movement analysis

Zebrafish larvae were recorded using a Blackfly S BFS-U3-31S4M dual color/infrared camera using Spinview software (Sony) in 48-well plates. Videos utilized a 4 million bitrate to ensure high-quality recordings. Larvae were tracked using Ethovision XT (V5) software with center point tracking total movement and circling behavior. Larvae were acclimated in the machine for 10 min, with two 5 min light recordings, followed by 5 min in the dark, followed by another two 5 min light recordings and a second 5 min dark recording. ‘Total distance’ and ‘rotation’ parameters are reported.

## Supplementary Material

10.1242/joces.263657_sup1 Supplementary information
