# Supplementary material for: The P4-phospholipid flippase Atp11a is required for maintenance of eye and ear structure in zebrafish
Source: J Cell Sci. 2025 May 22;138(10):jcs263657. doi: 10.1242/jcs.263657 (PMC12148042; doi:10.1242/jcs.263657)
Supplement: Supplementary information [file joces-138-263657-s1.pdf]

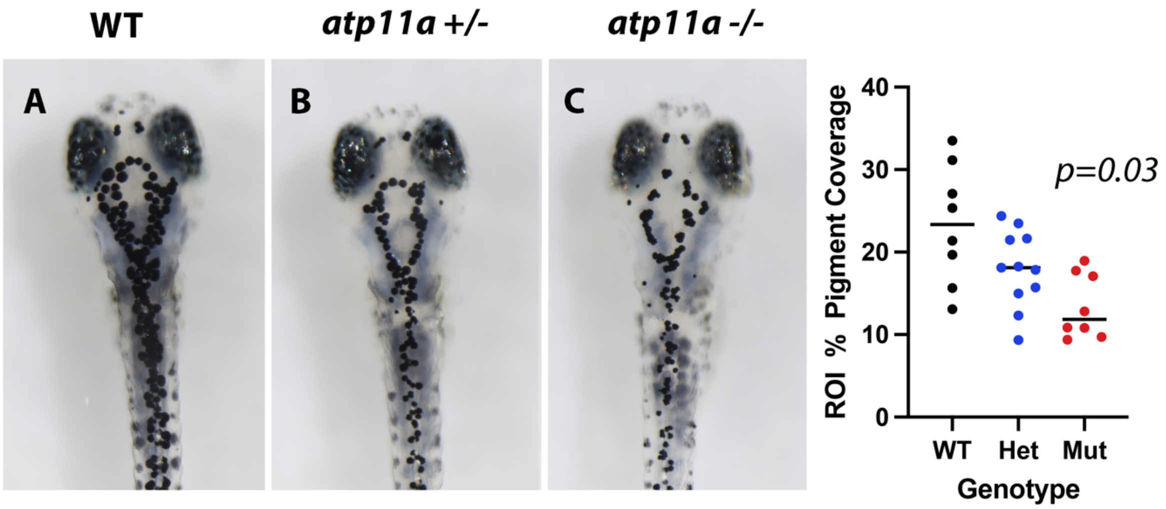

Fig. S1. *atp11a*^{nl1007} mutants are hypopigmented. Reduction in the pigment coverage in the head region of *atp11a* homozygous mutants when compared to wildtype siblings. Individual data points plotted with mean line shown. (WT) n=8, (Het) n=11, homozygous mutant n=8. Significance calculated using a one-factor ANOVA with post-hoc Tukey analysis for multiple comparisons. WT (n=8), het (n=11), mutant (n=8).

Journal of Cell Science • Supplementary information

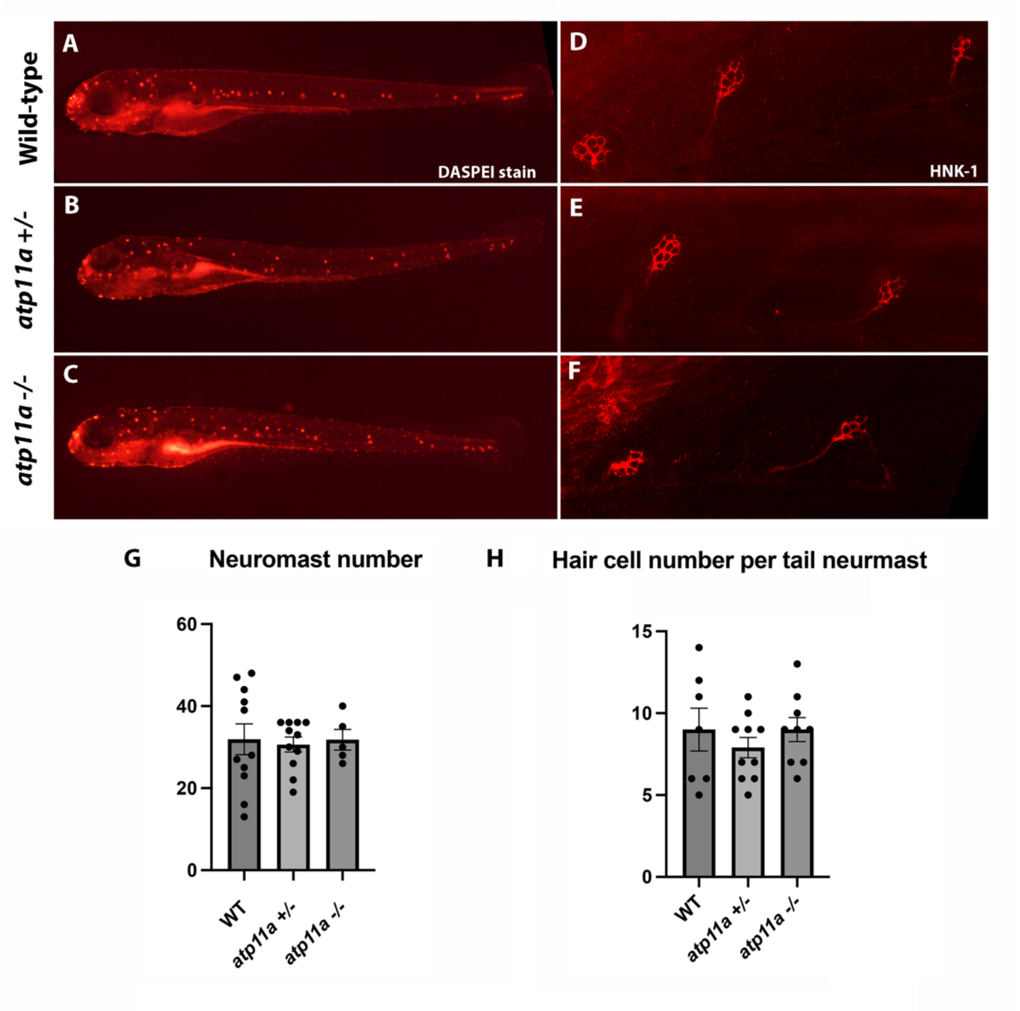

Fig. S2. No difference in neuromast number or innervation in *atp11a* mutants. **(A-C,)** DASPEI staining shows similar number neuromasts between Wild type, heterozygous and homozygous *atp11a*^{nl1005} mutants **(D-F)**, no difference in the intensity of staining of tail lateral line neuromasts is observed using the anti HNK-1 antibody between wildtype, heterozygous, or homozygous *atp11a*^{nl1007} mutants. (G) quantification of neuromast number in WT (n=11) heterozygous (n=11) and homozygous mutant larvae (n=5), presented as mean +/= SEM. (H) quantification of hair cell number in tail neuromasts via HNK-1 staining in WT (n=6), heterozygous (n=5) and homozygous mutant larvae (n=5), presented as mean +/= SEM.

Journal of Cell Science • Supplementary information

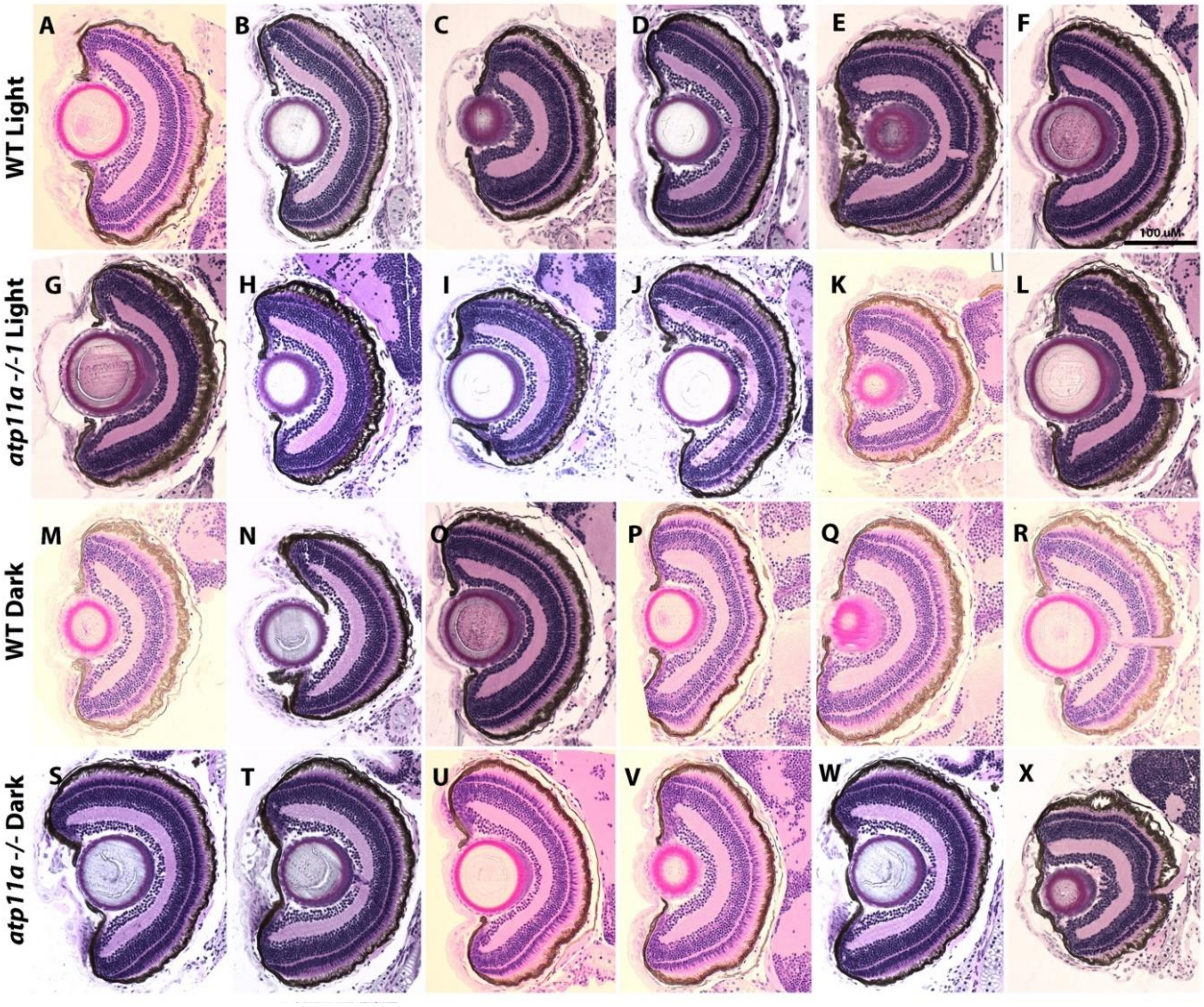

Fig. S3. Variability of photoreceptor outer segment loss in *atp11a -/-* mutants, and its rescue in dark raised larvae. Pictures are from at least two different larvae from three different experiments. (**A-F**) WT light raised larvae, (**G-L**) homozygous mutant light raised larvae, (**M-N**) WT dark raised larvae and (**S-X**) homozygous mutant dark raised larvae. Two larval sections displaying milder phenotypes in light raised homozygous mutants are shown in J and K.

Journal of Cell Science • Supplementary information

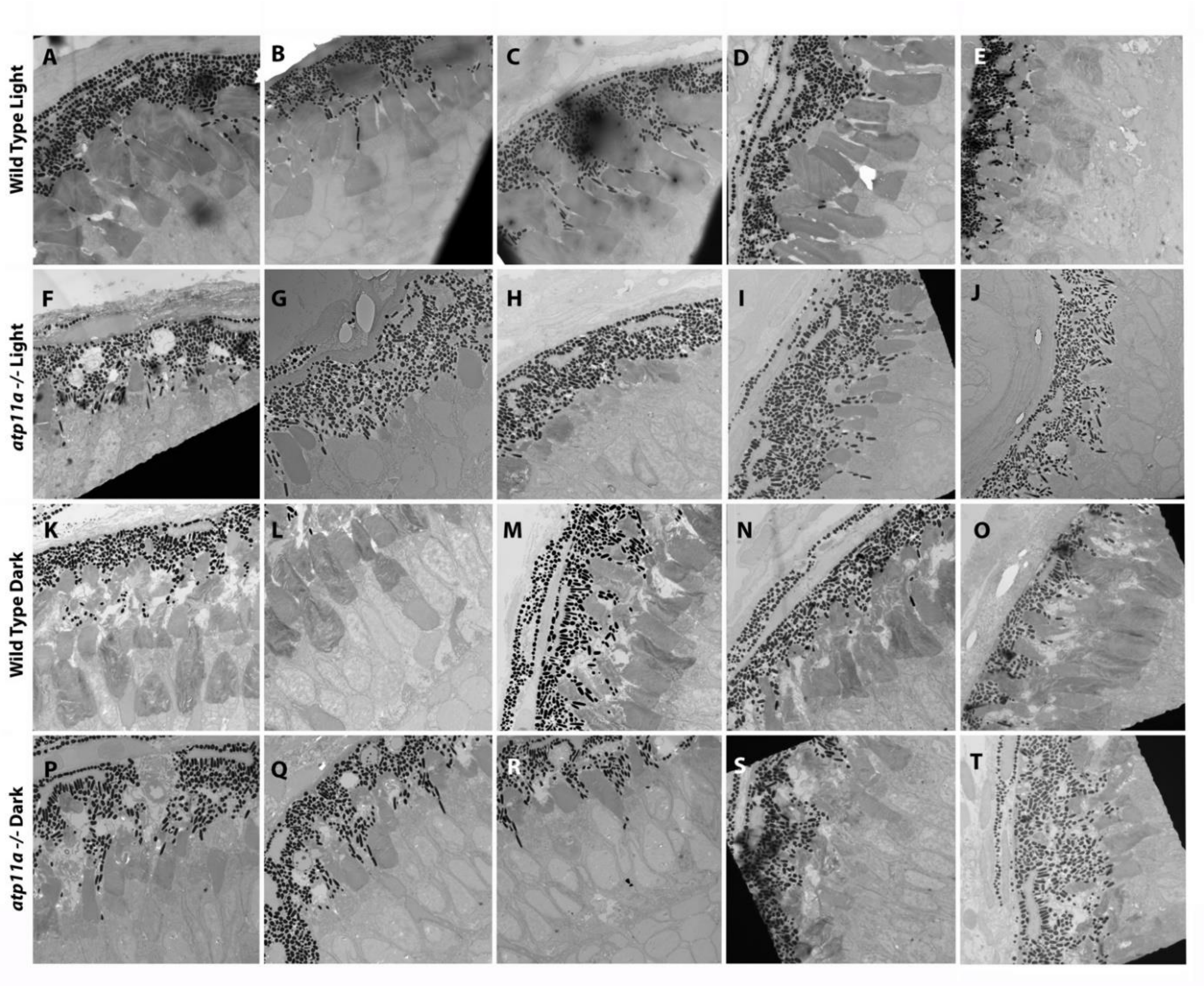

Fig. S4. Variability of photoreceptor outer segment loss as shown by TEM, and its partial rescue in dark raised larvae. Pictures are from at least two different larvae from two experimental groups. (**A-E**) WT light raised larvae, (**F-J**) homozygous mutant light raised larvae, (**K-O**) WT dark raised larvae and (**P-T**) homozygous mutant dark raised larvae. A larval section displaying a milder phenotype in light raised homozygous mutants is shown in (**I**).

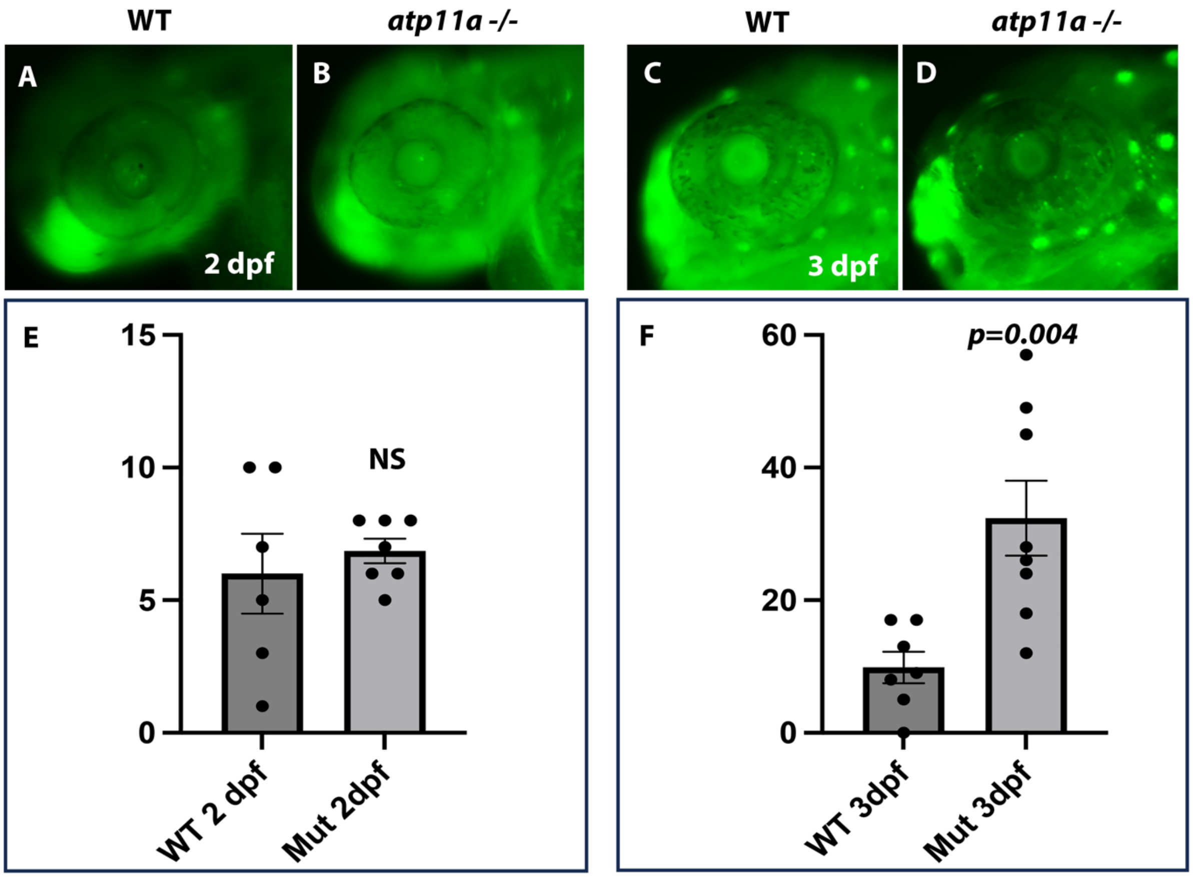

Fig. S5. Analysis of cell death in *atp11a*^{nl1007} mutants. (**A, B**) At two dpf, no change in the number of acridine orange positive cells is observed between wildtype and homozygous mutants (**C, D**) At 3 dpf a significant increase of acridine orange positive cells is observed in homozygous mutant eyes compared to wildtype siblings. (**E, F**) Quantification of acridine orange positive cells. Data presented as mean +/- SEM. WT 2 dpf (n=6), mutant 2 dpf (n=7), WT 3 dpf (n=7), mutant 3 dpf (n=8).

Journal of Cell Science • Supplementary information

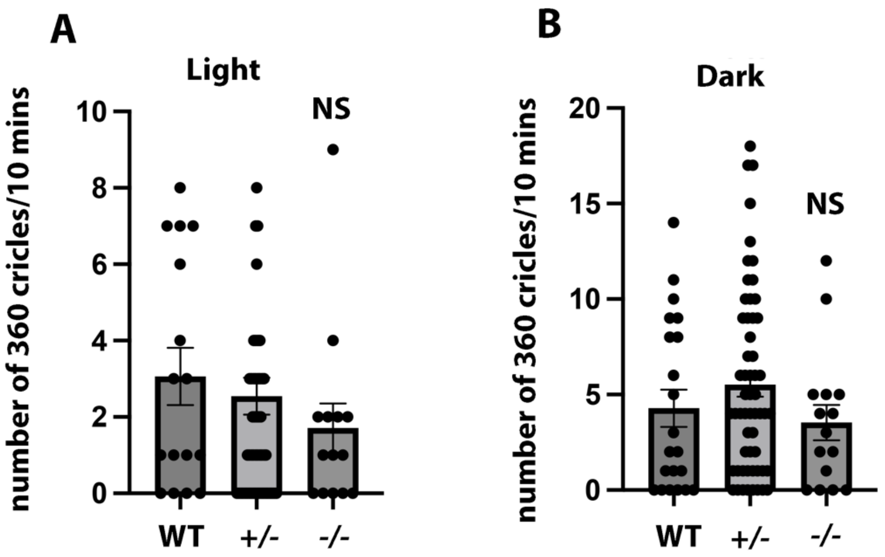

Fig. S6. Circling behavior in zebrafish clutches. (**A**) No difference is observed in homozygous *attp11a*^{nl1007} heterozygous or homozygous mutants in the light or (**B**) dark when comparted to wild type siblings. Data presented as mean +/- SEM. Significance testing via two-factor ANOVA. WT light (n=17) het light (n=57), mutant light (n=16), WT dark (n=22) het dark (n=57), mutant dark (n=15).

Journal of Cell Science • Supplementary information
